# Review of the Spatial Distribution, Source and Extent of Heavy Metal Pollution of Soil in China: Impacts and Mitigation Approaches

**DOI:** 10.5696/2156-9614-8.17.53

**Published:** 2018-03-12

**Authors:** Terefe Hanchiso Sodango, Xiaomei Li, Jinming Sha, Zhongcong Bao

**Affiliations:** 1 Fujian Normal University, College of Geographical Sciences, State Key Laboratory of Mountain Ecology, Department of GIS and Cartography, Fuzhou, China; 2 Fujian Normal University, College of Environmental Science and Engineering, Fuzhou, China

**Keywords:** soil, heavy metals, contamination, pollution, reclamation, remediation, bioremediation

## Abstract

**Background.:**

China has undergone a rapid industrial revolution and urbanization during the past three decades. This expansion is largely responsible for the release of a large amount of heavy metals into soils and is increasingly raising concerns over the potential effects on human health and the environment. The problem is drawing increasing attention, especially after an extensive nationwide soil survey report in 2014. A number of studies have examined soil contamination by heavy metals in China. However, most of these studies have been small in scale and it is therefore challenging to get a general overview of the level of contamination across the entire country.

**Objectives.:**

The present study is aimed at presenting a synthesized overview of the extent, pattern, and impact of heavy metal contamination of soils in China, including mitigation approaches.

**Methods.:**

Eighty-six journal articles and other literature such as reports, internet sources, and statistical yearbooks were narratively and critically synthesized to compile a holistic summary of sources of heavy metals, the extent of pollution, spatial distribution and impact of heavy metal contamination in China. The major findings from these studies are presented, along with mitigation approaches applicable to China.

**Discussion.:**

A synthesis of major findings from recent scientific journals shows that about 10.18% of farmland soils which supports 13.86% of grain production in China is affected by heavy metals. The main sources of pollution are anthropogenic activities. Even though the spatial distribution of pollution is highly variable owing to natural and human factors, provinces with intensive industrial activities such as Henan, Shandong, and Sichuan are more highly polluted than others. These regions are top grain producing areas and hence require close follow-up for development of feasible approaches to mitigating crop contamination and associated health risks emerging in parts of China. The government recently launched a program aimed at determining sound reclamation strategies.

**Conclusion.:**

Mitigation of heavy metal contamination in China requires coordination of different actors and integration of all feasible reclamation approaches.

**Competing Interests.:**

The authors declare no competing financial interests.

## Introduction

Soil contamination by heavy metal pollution is one of the most severe environmental threats to soil quality.^1^ The problem has received increasing attention in the last few decades in high- as well as low- and middle-income countries.^2^ The recent development of the global economy has resulted in an increase of heavy metals in soils, both in type and content.^3–12^ In particular, contamination of agricultural soils has increased due to the extensive use of chemical fertilizers, pesticides, livestock manures, irrigation waste water and sewage sludge, solid waste, agrochemicals and atmospheric deposition in attempts to increase crop yield.^5^,^10^,^13–15^

China has undergone rapid industrial revolution and urbanization, particularly during the past three decades. Industrial expansion has increased the amount of heavy metals released into soils.^3^,^6^,^8^,^10^,^12^,^16^,^17^ China feeds 22% of the world population with 7% of the worlds arable land.^18^ However, soil quality decline and associated ecological, environmental and health problems have worsened in recent years.^19^ This situation has alerted researchers, policymakers and the general public to the issue of sustainable soil usage.^20^ A nationwide extensive survey of soils conducted between 2005 and 2013 by the Ministry of Environmental Protection of The People's Republic of China (MEP) has attracted the attention of researchers and the issue has become an increasing concern for policy makers.^8^,^21^,^22^ According to the MEP, the total area of arable land polluted with heavy metals has reached 20 million hectares, accounting for approximately 16.1% of the total arable land in China.^23^ Even though the problem has been known to be severe for decades, it is getting worse and there are few feasible approaches to resolving the problem.^24^

## Methods

Scientific literature (written in English) focusing on heavy metal contamination of soils in China was searched and retrieved from journal databases, library catalogs, related websites, and reports. Greater focus was placed on recent peer-reviewed journals (published after 2000) focusing on the sources, extent, spatial patterns, and impact of heavy metal contamination of soils in China. Based on the findings of reviewed papers, feasible mitigation approaches for the context of China are discussed. All related references obtained from web search engines (Google web and Scholar, Yahoo and Baidu) were stored in a bibliographic management library (Mendeley software) and citations were retrieved from the database. Finally, the main findings of the related publications were narratively synthesized to provide a descriptive overview of heavy metal contamination in China.

## Study Area

**Figure 1:**
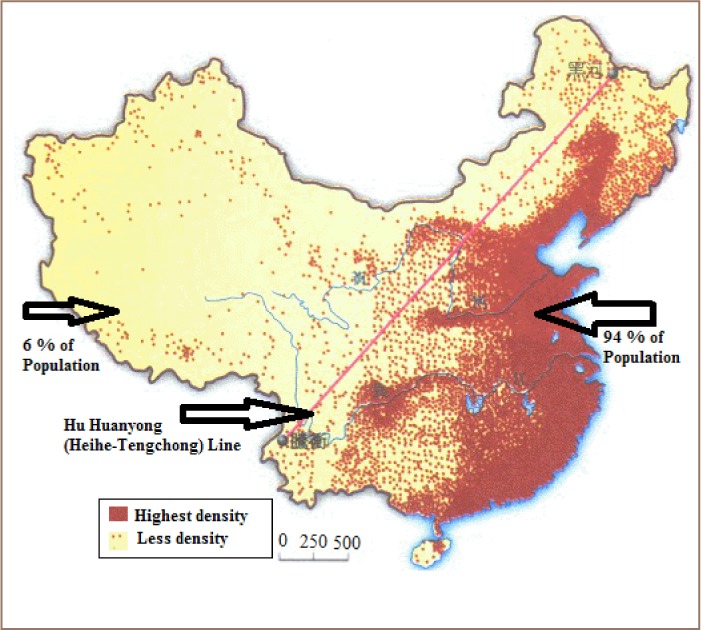
Mainland China with Hu Huanyong Line (Heihe-Tengchong line) 28

China is located in Southeast Asia along the coastline of the Pacific Ocean. It shares borders with Mongolia and Russia to the north, North Korea and the Pacific Ocean to the east, Vietnam, Laos, Burma, Bangladesh and the Pacific Ocean to the south, and Nepal, India, Pakistan, Afghanistan, Tajikistan, Kyrgyzstan, and Kazakhstan to the west, with a total area of around 9.597 million km^2^. The topography includes plateaus, plains, basins, and mountains. Nearly two-thirds of the land is occupied by rugged plateaus, foothills and mountains. China's broad plains are characterized by altitudes of over 500 meters and are dotted with foothills and low mountains. Broad plains are well-cultivated and are fertile crop-producing lands.^25^ According to the World Bank, the total population was estimated to be 1.378 billion in 2016.^26^ But the population density is lower in the west and higher in the East (greater than 300 persons/km^2^). According to Hu, et al. about 94% of the population lives to the east of the Hu Huanyong Line (Heihe-Tengchong line), an imaginary line that divides China into eastern (Manchuria and China proper-the 18 Han provinces) and the Tibet and Gobi Desert western region *(Figure 1)*. Average population density at a national level in 2013 was 143 person/km^2^.^25^,^27^

**Abbreviations**
*MEP*: Ministry of Environmental Protection of the People's Republic of China

Soil variation in China is complex due to the impact of climate, topography, vegetation and significant human influence.^29^ The zonal soil types in the eastern monsoon zone are latosol, lateritic red soil, red and yellow soil, yellow-brown soil, burozem and drab soil, dark brown forest soil, podzolic soil from south to north, and chernozem northeast to northwest, and chestnut soil, brown soil, sierozem, grey brown desert soil, and brown desert soil. On the Qinghai-Tibet Plateau, the soil types from east to west are alpine meadow soil, alpine steppe soil, alpine desert soil and frozen alpine soil. The soil of China is highly influenced by its long history of cultivation and agriculture, and also includes soil types such as paddy soil, oasis soil and lou soil.^29^,^30^

## Major Sources of Heavy Metals in Soil in China

Heavy metals in soil originate from many sources, including atmospheric deposition, sewage irrigation, improper disposal of industrial solid waste, mining activities, volcanic activities, the use of pesticides and fertilizers, and others.^3^,^7^,^10^,^31–33^ Sources of heavy metals in the environment can be categorized into natural and anthropogenic sources.^24^

**Figure 2:**
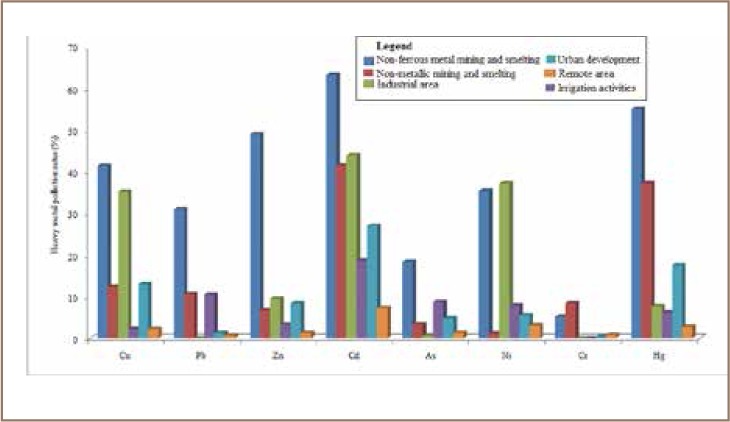
Heavy metal pollution rates across different sites in China adopted from Chen et al.^15^

Zhang *et al.* identified sources of heavy metals in arable soils in China with data from different studies. The study ranked non-ferrous mining and smelting activities as top pollution sources compared to agrochemicals in remote areas and urban and industrial sources (*Figure 2*).^15^

**Figure 3:**
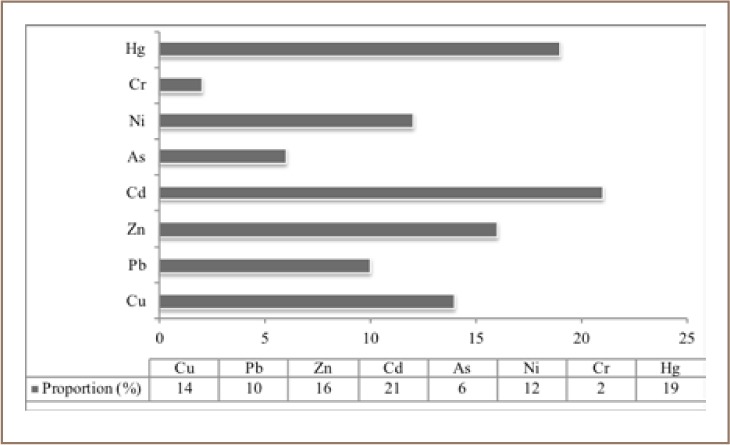
Contribution of non-ferrous smelting activities to heavy metal releases into the environment, modified from Zhang et al.^15^

Non-ferrous metal mining and smelting activities are the biggest contributors of heavy metal pollution as they result in large discharges of wastewater, waste gas and solid waste into the environment. Tan *et al.* described eight polluting industries including non-ferrous metal extraction and processing, non-ferrous metal smelting, oil exploration, petroleum processing, chemicals, coking, electroplating and tanning under the pointed title “China's Hateful Eight” and stated that these industries remained the major sources of heavy metal pollutions in 2017.^30^ Zhang *et al.* stated that compared to other elements, cadmium (Cd) is heavily released, causing very serious pollution, whereas chromium (Cr) is released in low amounts (*Figure 3*).^15^ In the same way, non-metallic mining and smelting activities that include coal and oil exploration, rare earth element and regular ore mining release high amounts of heavy metal elements into soils. Industrial activities including leather and cement factories also release high amounts of heavy metals into the environment. Other noticeable anthropogenic sources of heavy metals include irrigation activities, urban development activities, chemical fertilizers and pesticides.^15^

## Natural Sources

In China, the natural concentration level of heavy metals in soil is highly variable due to differences in soil type and soil parent material (soil genesis).^3^,^24^,^34^,^35^

Natural factors such as volcanic eruptions, degradation of minerals and forest fires, pedogenic processes, soil parent materials and evaporation from soil and water surfaces have been cited as the major natural sources of heavy metal pollution of soils.^3^,^24^,^34^,^35^ Some elements, especially the concentration of Cr and Mn, are correlated to natural factors such as soil genesis or parent material.^35^ Owing to the heterogeneity of soil types and parent material, the natural concentration level of heavy metals in the soil of China is highly variable.^3^,^24^,^34^,^35^ Additionally, in the tropical and subtropical regions of southern China, the acidic nature of the soils is one of the reasons for high accumulation of heavy metals.^36^ Natural sources are not the main sources of soil contamination by heavy metals except in Shanxi Province and the Inner Mongolia Autonomous Region where the concentration of As in the natural earth crust is high.^24^ Therefore, anthropogenic activities are the largest sources of the release of heavy metal elements into soils in China.

## Anthropogenic Sources

There are many anthropogenic sources of heavy metals concentrations in soil, but rapid industrialization and urbanization in China over the last three decades are considered to be the main contributors.^21^ Activities such as fertilization, agrochemical application and atmospheric deposition, sewage irrigation, mining, sludge application and smelting operations for metallic ores, industrial wastes, combustion of fossil fuel refining, and refinishing are some of the ways through which heavy metals accumulate in the soils.^2^ For instance, coal consumption in 2010 emitted approximately 9000 tons of arsenic (As), 360 tons of Cd, 450 tons of mercury (Hg) and 25000 tons of lead (Pb).^37^ Atmospheric deposition, which is considered to be the main source of heavy metal contamination of the atmosphere, pollutes at a greater rate in China than in any other developed countries.^38^ For instance, the atmospheric deposition of Cd ranges from 0.4 to 25 g ha^−1^ year^−1^, with a mean of 4 g ha^−1^ yr^−1^ in China.^37^ A study by Wu *et al.* inventoried anthropogenic emissions of mercury in China from 1995 to 2003 and estimated that Hg emissions from anthropogenic sources increased at an average annual rate of 2.9% during this period.^14^ Similarly, Zhong *et al.* identified the anthropogenic sources of Hg and their contributions in farmland soil and stated that activities such as the production of coke, application of fertilizers, discharge of wastewater, discharge of solid waste, and the production of non-ferrous metals were the main sources of Hg in farmland soil.^39^ The sources of heavy metals in urban soils and urban road dusts described by Wei *et al.* include traffic emissions (particles from vehicle exhaust, tire wear, weathered street surface and brake lining wear), industrial emissions (power plants, coal combustion, metallurgical industry, auto repair shops, chemical plants, etc.), domestic emissions, weathering of buildings and pavement surfaces, and atmospheric deposition.^10^ He *et al.* and Li *et al*. stated that the increasing production of electrical and electronic equipment is the driving factor for the large amounts of electrical wastes such as heavy metals (Cd, cobalt (Co), Cr, nickel (Ni), and Pb).^32^,^40^ The availability of large quantities of electrical waste in China remains a big challenge. He et al. stated that China had 69,520,200 sets of waste electrical and electronic equipment in 2011 alone, including 27,536,700 televisions, 7,611,000 refrigerators, 12,139,100 washing machines, 1,545,800 air conditioners, and 20,687,600 computers.^32^

Many studies confirmed the high correlation of industrialization and urbanization activities with an increase of soil heavy metals in China. For instance, according to He *et al.*, the production of Hg increased by 40% from 2004 to 2007, exceeding 1500 tons in 2007.^32^ Wu *et al.* estimated that total Hg emissions from all anthropogenic sources increased at an average annual rate of 2.9% from 1995 to 2003.^14^ Ying *et al.* determined the concentrations of As, Hg, Pb, Cd, Cr, and copper (Cu) in Huainan city, Anhui, East China. The study indicated a correlation between Cu, Cr, Cd, and Pb and industrialization and urbanization, as these metals mainly originated from automobile exhaust, coal gangue, fly ash, and industrial wastewater. However, As and Hg originated from coal combustion exhaust.^12^ Liu *et al.* also confirmed that industrialization and urbanization caused serious soil heavy metal pollution by conducting a combined analysis of the landscape pattern, urbanization, industrialization, and heavy metal pollution in Taiyuan city using multivariate analysis.^17^ Zhang *et al*. reviewed 465 published papers and confirmed that human activities such as mining and smelting, industry, sewage, urban development, and fertilizer application released heavy metals into the soil, which resulted in pollution of farmland soil.^15^

Similarly, Wang *et al.* conducted a field investigation to assess the status of heavy metal pollution in urban soils of Dushanzi, a district in Xinjiang, and analyzed the concentration of seven elements (Cu, zinc (Zn), Cd, Pb, Cr, As and Ni) and found that the mean concentrations of these metals were higher than corresponding background values. The study confirmed that the soils of Dushanzi were polluted by heavy metals and demonstrated a high ecological risk, especially for Cd. The study further stated that heavy metal pollution of urban soils in the area was mainly attributed to the petrochemical industry, coal chemical industry, traffic and commercial activities.^9^

However, according to Zhang *et al.*, the main sources of heavy metal pollution in rural areas include industrial waste from 1.6 million township enterprises which were relocated to rural areas due to lower land and labor costs. The study stated that wastewater discharged from these enterprises in rural areas was responsible for the pollution of 21% of all industrial wastewater in China. As per the same study, particulate emissions accounted for 13.2 million tons or 67% of total industrial emissions and there were 380 million tons of solid waste, accounting for 37% of industrial total solid wastes.^15^ For instance, according to the MEP, about 3,256.2 million tons of industrial solid wastes and 19.744 million tons of main pollutants were released in 2014 alone.^23^

Generally, human activities such as industrial emissions, wastewater and solid waste are the largest anthropogenic sources of heavy metal soil pollution in China.^2^,^3^,^10^,^17^,^24^,^35^,^41^ As the studies presented above illustrate, anthropogenic sources of heavy metals are largely responsible for environmental pollution in China in general, and soil pollution in particular.

## Extent of Soil Contamination in China

Heavy metal pollution of soil has been ignored for many years in China, but has garnered increasing attention after a nationwide soil survey report revealed how severely contamination is increasing and affecting sustainability.^41^ The MEP reported that the total area of arable land polluted with heavy metals has reached 20 million hectares, accounting for approximately 16.1% of the total arable land in China: 11.2%, 2.3%, 1.5%, and 1.1% with slight, mild, moderate, and heavy pollution levels, respectively.^23^ According to the report, a total of 19.4% of monitored cropland sites were above the normal range, and 13.7%, 2.8%, 1.8% and 1.1% had slight, mild, moderate and heavy pollution levels, respectively. The same report stated that about 10.0% of forest land, 10.4% of grassland and 11.4% of unused land sites were above the normal range. Inorganic pollutants were the major cause (82.8%) of pollution, exceeding the upper limits of national environmental standards. For the eight most common inorganic pollutants, monitoring sites had excessive concentrations of 7.0%, 1.6%, 2.7%, 2.1%, 1.5%, 1.1%, 0.9% and 4.8% for Cd, Hg, As, Cu, Pb, Cr, Zn, and Ni, respectively.^23^,^42^,^43^

**Figure 4:**
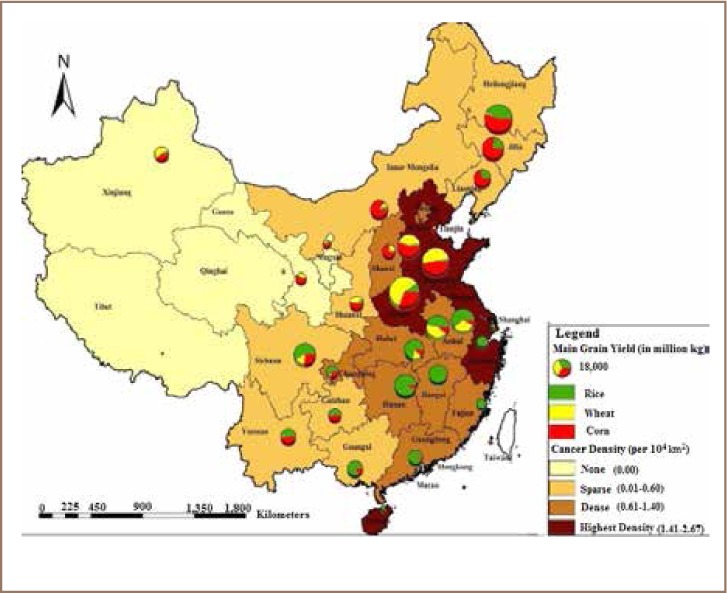
Distribution of cancer villages and main grain yield in China^44^

Lu *et al.* defined a ‘cancer village’ as a village where the morbidity rate of cancer is significantly higher than the national average.^44^ The author associated cancer villages with heavy metal pollution rate in China and assessed the correlation. The study showed the association between cancer village density and important grain producing areas that have been affected by heavy metal pollution. As shown in Figure 4, cancer villages are concentrated in the eastern parts of the country where there is very high grain production, intensive agricultural practices, urbanization and associated soil contamination, showing a clear pattern of variation between western and eastern China. The eastern sea coast areas had the highest density of cancer villages, indicating that there may be high pollution in these areas due to high urbanization, leading to increased contamination by heavy metals. The study has recommended the use of an integrated approach to combating soil and water contamination and its associated health impacts.^44^

Some studies have reported that the geological background level of heavy metals in soils of China is low.^10^ Similarly, Zhao *et al.* claimed that the current status of soil contamination based on total contaminant concentrations in China is on par with other regions of the world.^21^

In contrast, the majority of studies have found that the concentrations of some heavy metal elements are higher than the recommended levels of contaminants in food crops, especially in southern China. Moreover, the increase has been swift over the last two decades owing to rapid growth of the economy, industrialization and anization.^3^,^6^,^8^,^10^,^12^,^14^,^16^,^21^,^24^,^31^,^32^,^34^,^35^,^40^,^45–48^ Even though the seriousness of the problem has been known for the last few decades, pollution continues to worsen and there have been limited successful approaches to resolving this issue.^24^ However, due to the emergence of comprehensive pollution reports in 2014, greater focus has been given to the extent of emissions of Pb, Hg, Cd, Cr and As due to their high toxicity, prevalence, and persistence in the environment.^23^ Similarly, many studies have focused on these heavy metal elements mainly to determine the extent of their emissions on smaller spatial and temporal scales. For instance, Cheng *et al.* assessed contamination by heavy metals (As, Cd, Cr, Cu, Hg, Ni, Pb, antimony (Sb), selenium (Se), and Zn) in urban soils across 31 larger cities in China and stated that in terms of geo-accumulation, Hg, Cd and Se were the biggest soil contaminant metals in China's urban areas as Hg and Se are emitted from fossil fuels.^3^ Likewise, Zhang *et al.* reviewed 465 published papers to evaluate the rates of heavy metals pollution in farmland soil and found that Cd had the highest pollution rate, followed by Hg, Cu, Ni and Zn and Pb, whereas Cr had the lowest pollution rate. The study stated that the total pollution rate was highest in farmland soil, with pollution mainly attributable to Cd, Hg, Cu, and Ni elements affecting significant amounts of grain production.^15^ Ying *et al.* determined that the average concentration of As, Hg, Pb, Cd, Cr, and Cu in Huainan city, Anhui, eastern China was high.^12^ Chen *et al.* carried out a study in northeast China in Fuxin City which is known for resource overexploitation and conversion to industry. The study confirmed that concentrations of As, Cd, Cr, Cu, Ni, Pb and Zn were very high due to human activities such as mining, industrial production and agriculture production.^16^ Li *et al.* determined Cr, Cu, Zn, Pb, Cd, As, and Hg concentrations in surface sediment of Dongting Lake and reported that the mean contents of As and Cd exceeded the probable effect level by 58% and 50%, respectively.^34^ Peng *et al.* determined the means and ranges of heavy metal concentrations in sediments, soils and water along the Shunde waterway and estimated the hazard quotients of heavy metal species.^47^ The study confirmed that As and Cd were the major pollutants contributing to health risks in the area through vegetable uptake and leaching to groundwater. In similar way, Zhang *et al.* determined the concentrations of selected heavy metals (Cu, Pb, Zn, Cd, Cr, Ni and iron (Fe)) in western Xiamen Bay.^49^ The study confirmed that Pb contamination exists throughout the entire study area and contamination of other metals is also present in some locations. Zhuang *et al.* also investigated the levels of Cd, Pb, Cu and Zn in the environment and food sources in the Dabaoshan mine area in southern China and estimated potential health risks.^48^ The study confirmed that Cd, Pb, Cu and Zn concentrations in arable soils near the mines exceeded standard values. The concentrations of Cd and Pb in some food crop samples were significantly higher than the maximum permissible levels. Wang *et al.* conducted a field investigation in urban soils of Dushanzi, a district of Karamay city in Xinjiang and analyzed concentration of seven elements (Cu, Zn, Cd, Pb, Cr, As and Ni).^9^ The study confirmed that urban soils in Dushanzi were polluted by heavy metals and demonstrated a high ecological risk. Likewise, Li *et al.* investigated the status of heavy metal soil pollution in the Tiexi Industrial District in the city of Shenyang, and found that the concentration of Pb, Cu, Cr, Zn, Mn, Cd, As and Hg were higher than the background values.^35^

It can be concluded that even though the background values of heavy metals in the soils of China are low, anthropogenic activities have contributed to an alarming increase of heavy metal concentrations and pollution in the soils across China.

Even though the pollution rates for arable soil vary depending on the spatial and temporal scope of different studies, most of the findings estimate the farmland pollution rate to be 10.18%, mainly from pollution from Cd, Hg, Cu, and Ni.^15^ Studies of farm soil pollution by Cu, Pb, Zn, Cd, As, Ni, Cr and Hg found the average concentration of toxic metals to be 3.01 mg Kg−1 for Cu, 0.96 mg Kg−1 for Pb, 2.09 mg Kg−1 for Zn, 7.75 mg Kg−1 for Cd, 1.54 mg Kg−1 for As, 2.88 mg Kg−1 for Ni, 0.60 mg Kg−1 for Cr, and 3.65 mg Kg−1 for Hg.^15^,^23^,^24^

## Spatial Distribution of Heavy Metal Contamination in China

**Figure 5:**
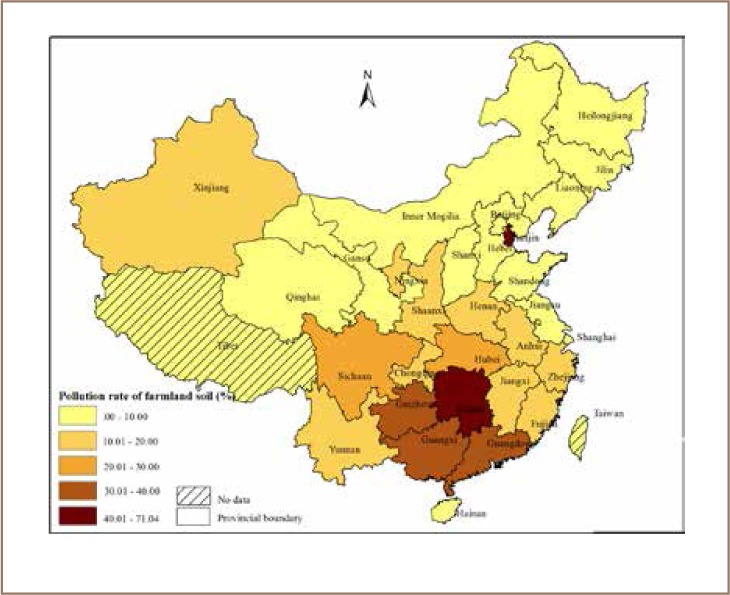
Farmland heavy metal pollution rates by province.^15^

Many studies have assessed the spatial distribution of heavy metals in the soils of China.^4^,^6^,^8–10^,^12–14^,^16^,^21^,^24^,^31^,^32^,^34–36^,^41^,^45–48^,^50^ The distribution of heavy metal pollution has high spatial variation in China^24^ due to complex natural and anthropogenic factors that vary across different regions of the country. For instance, cities with high Hg pollution levels are found throughout the northeast and southeast of China, mainly in the Liaohe River and Yangtze River watersheds where gold mining, chemical industries, and coal combustion are important anthropogenic sources.^51^ However, cities with high Pb concentrations are concentrated upstream of the Yellow River, Yangtze River drainage areas, and the Pearl River basin.^51^ Pollution may follow the Hu Huanyong Line (Heihe-Tengchong line) that divides China into eastern and western regions. The eastern region has 36% of the land area, but around 96% of the total population. Additionally, the distinctions created due to differences in urbanization, industrialization, economic development and population density between the western and eastern regions may have an influence on the spatial variation of heavy metal pollution levels. The spatial distribution of soil contamination by heavy metals also shows an increasing trend towards the eastern regions of the country. Similarly, the eastern provinces had a higher farmland soil contamination rate compared to the western provinces, except for Xinxiang province, which had a relatively higher pollution rate than other counties in western China (*Figure 5*). Likewise, Hunan, Henan and Sichuan provinces had the highest amount of grain production affected by heavy metals compared to other provinces. Hebei, Anhui and Shandong provinces in the eastern part of the country also had a higher proportion of grains affected by heavy metals.^15^

According to the MEP, fourteen key provinces make up the biggest share of the country's heavy metal discharges, including Pb (90%), Hg (88%), Cd (93%), Cr (74%) and As (84%).^30^ Tan *et al.* reported that over 70% of farming provinces were included in the priority list, accounting for 98 million hectares or 60% of China's cropland. That means that only 40% of China's cultivated land is on the non-priority list for heavy metal pollution.

**Figure 6:**
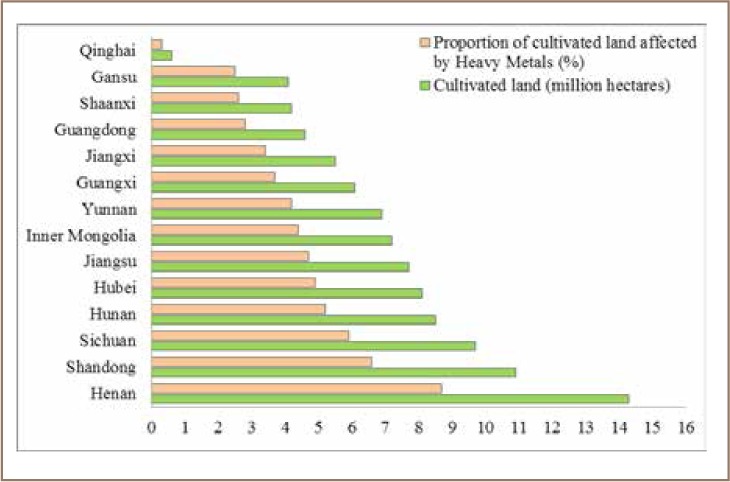
Proportion of cultivated land in 14 provinces identified with high heavy metal discharges, adopted from^23^

For instance, in 2012, Hunan alone was responsible for 39% of Pb, 50% of Cd, and 42% of As discharged into the environment. The fact that 13% of China's total rice production comes from Hunan province, which produced a total of 26 million tons of rice in 2012 alone, raises a major concern for food safety. As indicated in Figure 6, Henan province has highest proportion of cultivated land followed by Shandong and Sichuan, which were also reported to be highly affected by soil contamination.^23^

However, as discussed above, it is difficult to find comprehensive and nationwide heavy metal distribution studies. Most of the existing studies employ narrow spatial and temporal scales. A summary of some of the studies focused on the spatial distribution of heavy metal pollution in China are shown in Supplemental Material 1. Most of these studies confirmed the availability of a significant amount of major heavy metals such as As, Cd, Cr, Cu, Hg, Ni, Pb, and Zn in the soils. Lead was the most examined element in the reviewed studies that determined the concentration of heavy metal elements in soils (Supplemental Material 1). Cu was the second most studied element, followed by As. Other elements which were not listed in the table were not commonly studied in the current reviewed literature in this study.

Some studies calculated the ecological toxicity and spatial distribution of these metals, whereas others focused solely on determining the concentration of heavy metals and extent of pollution.^16^,^35^ For instance, Chen *et al.* determined the spatial distribution of As, Cd, Cr, Cu, Ni, Pb, and Zn in surface soils (0–20 cm) in industrials areas of northeastern China.^16^ The study found that all of the investigated metals showed distinct geographical patterns, with higher concentrations in urban and industrial areas than in farmland. Cheng *et al.* confirmed that the type and concentrations of trace metals varied from city to city.^3^ Li *et al.* determined the extent and spatial distribution of soil pollution in the Tiexi Industrial District in the city of Shenyang.^35^ The study identified hotspot areas of high metal pollution using geostatistical analyses. They confirmed that Pb, Cu, Zn, Cd and As were more highly correlated to anthropogenic activities, especially industrial processes. However, Cr and Mn concentration patterns had low spatial heterogeneity across these activities, indicating a low correlation between Cr and Mn and human activities, suggesting that their levels are instead affected by natural factors such as parent soil materials.

Wu *et al.* studied regional Hg emission trends for provinces in China from 1995 to 2003.^14^ The study revealed significant differences in trends of total Hg emissions across provinces; for instance, Ningxia and Guangdong provinces showed higher Hg emission growth over the national average during the survey period; however, Xinjiang and Heilongjiang provinces show reduced Hg emissions over the same time. The study further related increased Hg emissions in some provinces with increased consumption of coal in the industrial sector and zinc smelting. However, power-related (lamp, battery, florescent) Hg emissions were the biggest sources of the increase of Hg in Fujian Province.

**Figure 7:**
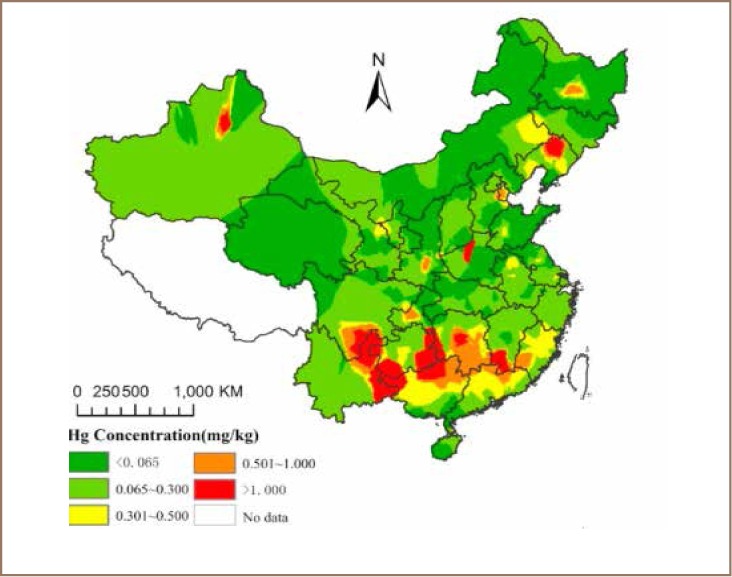
Spatial distribution of estimated Hg concentrations in agricultural soil in mainland China^52^

Cheng *et al.* and Wu *et al.* also stated that spatial distribution of Hg concentrations in agricultural soil in China is influenced by natural factors and anthropogenic activities*(Figure 7)*.^3^,^14^The study identified several Hg hotspots in Hunan, Guangxi, Guizhou, Yunnan, Sichuan provinces owing to Hg, molybdenum, Sb, and Pb or Zn mining and smelting activities in Liaoning, Heilongjiang and Jiangxi, coal mining in Henan, and sewage irrigation in Xinjiang. It stated that mercury mining areas were hot spots of Hg pollution because cinnabar ore roasting generates huge quantities of mine waste. Moreover, the same study found that Hg concentrations in the soils of Hg mining areas in Guizhou province reached 150.00 mg/kg, about 500 times the grade II reference in agricultural soil. Discharge from Sb, Pb or Zn, molybdenum, coal mining and related activities could also lead to large amounts of Hg, along with wastewater, waste gas and solid waste being added to the environment.^3^,^14^

According to Cheng *et al.*, there has been a steady rise in the emission of heavy metals and other pollutants in urban areas due to increasing population and urban growth.^3^ The study also claimed that the contamination was not only confined to urban areas, but is widely dispersed in surrounding areas in the soil, atmosphere, water, and plants. Urban soil is more affected due to the high anthropogenic influence in the form of industrial, commercial, and domestic activities. The study further stated that the cities of Shanghai, Kunming, Shenyang, and Changsha were more seriously affected than others. Moreover, high concentrations of metals in soils were found in Changsha, Shanghai, and Shenyang due to the presence of metallurgical industries and smelt mining.

Similarly, He *et al.* evaluated the spatial distribution of heavy metals pollution in selected cities.^24^ The study confirmed that Guizhou, Guangdong, Shanxi and Liaoning provinces had the highest levels of Hg contamination. The reason for the high level of Hg in Guizhou Province is because the province is one of the world's biggest producers of Hg owing to its geochemical characteristics and anthropogenic activities. Soil around an industrial park in Xiangtan, Hunan province was also found to be a hot spot for Cd pollution due to mining activities.^24^ Likewise, Zhang *et al.* evaluated the spatial distribution of Cd in arable soil and identified hotspots in Liaoning, Guangxi, Guangdong, Hunan, Yunnan, Gansu, and Henan Provinces.^53^ The Guangxi Zhuang Autonomous Region had the most hotspots, and it connects to the hotspots of Hunan, Guangdong, and Yunnan Province.^53^

He *et al.* stated that China is highly affected by Cr waste as it is the biggest producer of Cr waste in the world owing to very high production and consumption of leather products in the country.^24^ Likewise, the presence of arsenic has been frequently reported over the past several decades, especially in Xinjiang Uygur, Inner Mongolia and the Ningxia Hui Autonomous Regions and Shanxi Province. China is the world's largest coal producer with high levels of coal combustion, especially in Guizhou Province, and this practice has been reported to cause arsenicosis. The same study reported high amounts of As collected from coal mines and local consumption in open ovens in northeastern, northern and eastern China, which carries a risk of pollution to indoor air and food. Additionally, human activities such as mining and acid production are sources of serious As pollution around abandoned tungsten mines and industrial districts. Similarly, Pb concentrations in farmland soils near a highway were found to be high, ranging from 0.0001 to 0.00018 mg m^−3^. Soils (farmland) on the sides of the highway are more vulnerable to Pb pollution due to traffic emissions, and soils near the highway are more polluted by Pb than soils further away from these emissions. Different land uses result in varying concentration levels of heavy metal elements. For instance, He *et al.* found that park areas had lower Pb levels than roadside soil, and industrial areas had higher concentrations than residential areas. Lead concentrations in farmland soil irrigated by wastewater were also increased.^24^

**Figure 8:**
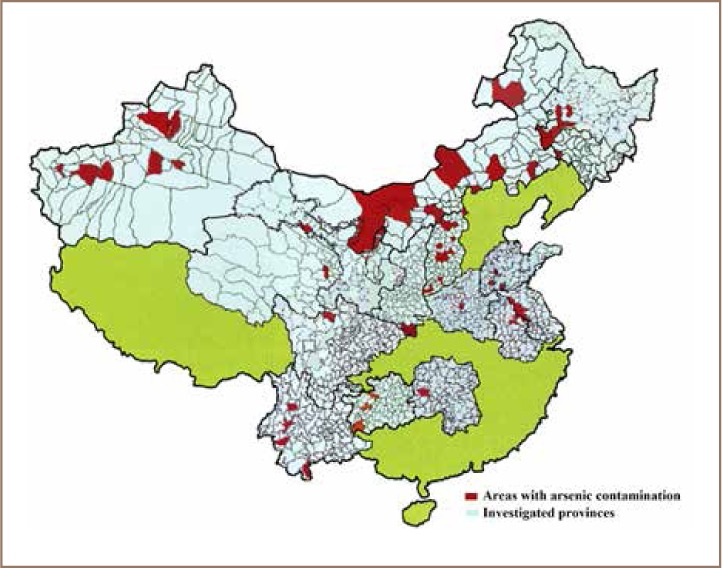
Areas with high levels of As pollution in China, 2006. 54

Moreover, according to Sun *et al.*, the pollution of water sources with As is severe based on a nationwide survey of arsenicosis carried out in mainland China between 2001 and 2004.^54^ It is known that inland water bodies and farmland soils are very much interlinked. Additionally, inland water is frequently used for drinking water and irrigation of farmland, and therefore heavy metals are easily transported to soils and crops. This leads to heavy metal transfer to humans through water-soil-plant transfer and drinking water. Soil, water and plants are interconnected in analyses of heavy metal contamination and hence water is an important potential source of arsenic exposure for humans. Even though the national maximum acceptable concentration for arsenic in drinking water is five times (i.e. 50 mg/L) the World Health Organization recommendation (10 mg/L), the concentration level in different provinces of China was found to be higher. The provinces of Shanxi, Qinghai, Sichuan, Inner Mongolia, Jilin, Xinjiang, Gansu, Anhui, Jiangsu, Ningxia, Henan, Heilongjiang, Yunna, Shandong, and Hunan were found to have elevated levels of arsenic in water.^54^ Moreover, Xinjiang, Inner Mongolia, Shanxi, and Guizhou provinces were identified as arsenicosis epidemic areas, with districts such as Jilin, Ningxia, and Qinghai emerging as new epidemic areas. According to Sun, *et al.*, arsenicosis was found to be epidemic in a total of eight provinces and 37 counties in mainland China.^54^ Unlike other toxic metals that have higher concentrations in populated, urbanized and developed areas in eastern China, the spatial distribution of As is dispersed across mainland China without a clear pattern *(Figure 8)*.

## Impact of Heavy Metal Pollution in China

### Economic Loss

According to the MEP's report, about 20 million hectares of land is contaminated, accounting for an annual loss of about 12 million tons of grains (mainly rice and other crops) contaminated by heavy metals. This contamination alone results in an annual economic loss of 20 billion renminbi (RMB) (3.3 billion USD). For instance, Hunan province is China's largest rice producer, producing about 26 million tons of unhusked rice, accounting for nearly 13% of China's total in 2011. However, the province is also among the top five producers of nonferrous metals such as Cd and Pb due to the large number of mines (7.5% of the country's total).^55^ As a result, a high proportion of Cd tainted rice reported to be from Hunan province has been found in the market.^55^

### Food Contamination

The combined effects of industrialization, urbanization and recent rapid economic growth in China have led to direct or indirect heavy metal contamination through agricultural soils, food, water, and air.^4^,^8–10^,^24^,^41^,^50^ Consequently, a large proportion of the population has been exposed to contaminated agricultural or urban soils in the form of food crops (rice, vegetables, fruits, etc.), skin absorption, and inhalation of dust, resulting in a variety of health problems.^7^,^8^,^12^,^13^,^40^,^48^ Zhao *et al.* argued that residents living in contaminated areas who consume mainly locally produced grain and vegetables are vulnerable to health risks.^21^ Furthermore, Zeng *et al.* analyzed Cd, Cr, As, Ni, Mn, Pb, and Hg in three agricultural areas of Hunan province and determined that concentration levels of Cd and Hg were highest, followed by As and Ni.^56^ The mean concentrations of heavy metals in brown rice were 0.325, 0.109, 0.344, 0.610, 9.03, 0.023, and 0.071 mg/kg, for Cd, Cr, As, Ni, Mn, Pb, and Hg, respectively. The study reported that Cd and Hg had greater transfer ability from soil to rice than the other elements. The study further determined the daily intake of heavy metals from brown rice consumption to be 0.0023, 0.000775, 0.00245, 0.00432, 0.000162, 0.0646 and 0.000503 mg/(kg · day) for Cd, Cr, As, Ni, Pb, Mn, and Hg, respectively.

### Health Impacts

Tchounwou *et al.* stated that heavy metals toxicity in humans depends on the dose, route of exposure, and chemical species, as well as the age, gender, genetics, and nutritional status of the exposed person.^8^ Human exposure to heavy metals through the food chain could lead to dangerous health risks owing to the non-biodegradable nature of these trace elements in the human system. He *et al.* categorized health disorders according to target organs affected into renal disorders, gastrointestinal effects, neurological effects, cancer, and others. Overall, studies have confirmed that the health effects of heavy metal contamination are varied and complex.^24^

Among heavy metals, As, Cd, Cr, Pb, and Hg are the most important toxicants due to their high degree of toxicity and public health significance.^6–8^,^13^,^36^,^40^,^41^ These metals have the capability to damage multiple organs with small exposures, although very small amounts of Cu, Zn, and Ni are needed in the human body.^7^,^8^,^12^ In the same way, high levels of arsenic contamination can cause a number of human health effects. Several studies have reported a strong relationship between arsenic exposure and high risks of carcinogenic and systemic health effects.^8^,^12^,^56^ According to Qingjie *et al.*, arsenic exposure affects all of the human organ systems, including the cardiovascular, dermatologic, nervous, hepatobiliary, renal, gastrointestinal, and respiratory systems.^2^ In recent years, many studies have focused on arsenic as a large proportion of the population in China has been exposed to high concentrations of arsenic. This exposure has led to risks of cardiovascular and vascular diseases, growth abnormalities, neurologic disorders, diabetes, hearing loss, portal fibrosis, hematologic disorders and carcinoma across China.^7^,^8^,^16^,^24^,^41^

**Figure 9:**
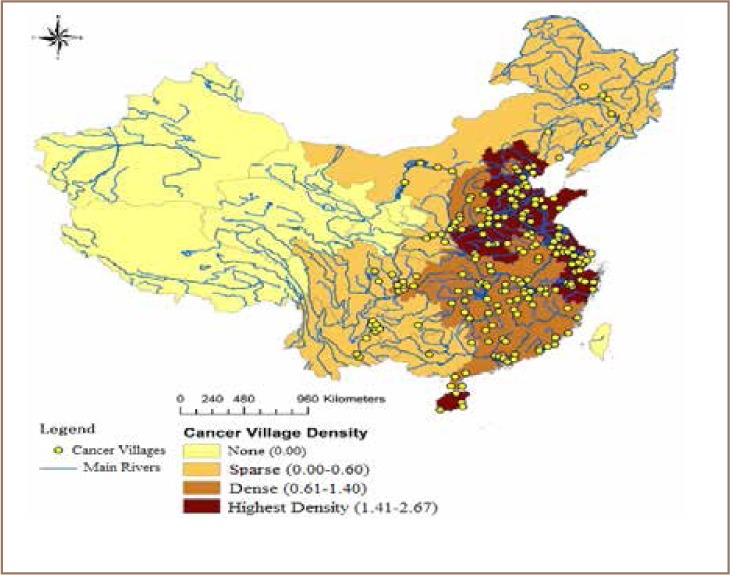
Soil contamination risk and cancer villages.^44^

Lengthy exposure to these heavy metals should be avoided to prevent health risks, and even very small exposures may impact the health of children. Chao *et al.* found that in about 30% of children's blood samples, the content of Pb exceeded the residential standard (100 gm/L), and in cities, this rate increased to over 60%.^57^ Zhao *et al.* argued that protecting agricultural soils (rice fields) from contamination of Cd, Pb and other metals can help to mitigate impairment to immunological defenses, psycho-social faculties, and disabilities associated with malnutrition.^58^ Chao *et al.* reported that workers who were in close contact with nickel powder were more likely to suffer from respiratory cancer, and the content of Ni in the environment was positively correlated with nasopharyngeal carcinoma.^57^ According to Lu *et al.,* most of the cancer villages are clustered in eastern China (*Figure 9*), an area that is widely known for high grain production and high population density.^44^

### Ecological Impacts

Heavy metal pollution affects not only crop production and health, but also the quality of the atmosphere, waterbodies, climate and ecosystems.^34^,^36^,^59^ Tang *et al.* argued that the aquatic ecosystems in eastern China suffer from heavy metal pollution.^59^ The study determined heavy metals in surface sediments from different aquatic ecosystems (river, reservoir, estuary, lake, and wetland ecosystems) and found the average concentrations of Cd, Cr, Cu, Ni, Pb, and Zn to be 0.716, 118, 37.3, 32.7, 56.6, and 204 mg/kg, respectively. These levels are higher than internationally acceptable limits for all metals. The study concluded that all five types of aquatic ecosystems were polluted with heavy metals, and the most polluted ecosystems were rivers. The study stated that Cd was the most serious pollutant in all five aquatic ecosystems. Furthermore, many studies confirmed that mining activities in northeast China have resulted in soil quality degradation and a decrease in surface soil porosity and available phosphorous. This has affected the survival of soil microbes and phyto-availability of nutrient elements in soil and this in turn has triggered ecosystem changes.^16^,^36^ According to Tan *et al.*, major rivers, lakes, and reservoirs are subject to different degrees of heavy metal pollution. The sediment contamination rate has reached 80.1% in some areas, seriously affecting the quality of water and aquatic life.^30^ For instance, the river sediment of China's largest river, the Yangtze, was found to have an excessive concentration of mercury and direct discharges of copper, zinc, and lead, polluting the surrounding environment and water bodies.^43^ The Pearl River, the second largest river in China, is reported to be affected by heavy metal pollution. According to Wang *et al*., the concentration of heavy metals in the river estuary has increased in recent years, and rates of Cu, Pb, Cd, As, and Hg were 80.77, 105.88, 5.55, 33.13, 0.33 mg/kg, respectively, well over the international limits of 0.005 mg/kg, 0.015 mg/kg, 0.005 mg/kg, 0.01 mg/kg and 0.002 mg/kg for Cu, Pb, Cd, As and Hg. The effects of heavy metal contamination include ecosystem disorder, biodiversity loss and climate change.^43^

## Mitigation Approaches

Various methods have been employed globally to remediate soils contaminated by heavy metals. These include physical, chemical and biological methods. Most of these methods, such as encapsulation, solidification, stabilization, electrokinetics, vitrification, vapor extraction, and soil washing and flushing have high costs. However, biological (bioremediation) approaches that involve planting on polluted soils are less expensive and are environmentally friendly. 60,61

In recent years, the government of China established a strong policy framework to mitigate heavy metal pollution of the environment, and prioritized minimizing the release of harmful contaminants.^21^ To that end, mitigation of emissions and close monitoring of sources of heavy metals such as mining, smelting and other metal-consuming industries are being implemented. Furthermore, a comprehensive monitoring framework was launched in 2014 to support research institutions and to implement sound reclamation strategies. According to the report, contaminated soil sites have been identified and a number of reclamation activities have been launched over the past few years and are expected to greatly benefit China's economy. It is estimated that reclamation of land through treatment or remediation could generate 200 billion yuan in annual revenue by 2025.^30^

Even though there are promising approaches, soil reclamation is an extremely difficult process, requiring large investments of time and technology. This is partly because heavy metal contamination affects not only the initial sites where contamination is prevalent, but may also spread to surrounding areas, through movement of soil particles from sources of pollution by wind, rain and other agents.

Moreover, even though there are several options for treating or cleaning up soils contaminated with heavy metals, it is extremely difficult to find one perfect method for all situations. There are many commonly used methods available for reducing the harmful effects of heavy metal-contaminated sites, including excavation (physical removal of the contaminated material), stabilization of metals in soil on-site, and the use of plants to stop the spread of contamination or to extract the metals from soil (phytoremediation), and others.

### Soil Excavation and Engineering

Soil excavation is the method most commonly used to treat contaminated soil in China. This approach involves removing contaminated soil and off-site treatment or disposal through excavation, capping and washing or burning of soils, requiring heavy machinery to excavate and transport soils to treatment sites.^60^ Treating soil ex-situ is expensive, whereas in-situ treatment using phytoremediation, phosphate addition and others methods can reduce costs.^60^,^62^

### Chemical Remediation (On-site and/or In-situ Precipitation)

Precipitation is a process used in wastewater treatment of heavy metals that involves conversion of a solution into a solid state through a change in the chemical equilibrium relationship between the solute and solution. In this process, the solubility of heavy metals is altered through reactions with specific chemicals, causing it to precipitate from the solution.^63^ This method can be adapted to soils where it can reduce the mobility of heavy metals. The on-site precipitation process includes chemical treatment with excavated soils, while in-situ precipitation involves application of chemicals directly to the soil to decrease metal mobility. There are four reduction/precipitation methods: the sulfide, sodium borohydride, cellulose xanthate and lime/carbonates/hydroxides processes. However, the application of these processes to soils contaminated with metals has not been studied in great detail. The applicability of this approach largely depends on variation in soil type, structure, texture, moisture and the extent of the contamination.^63^ This approach could be adopted depending on the extent of contamination and suitability of particular sites for chemical treatment in the context of China.

### Land Use Management and Agricultural Monitoring

A few recent studies have reported the effect of land use on soil contamination by heavy metal elements and confirmed that effective land use management is crucial for remediation of contaminated soils.^64^,^65^ Elrashidi *et al.* investigated the effects of four land uses (no-till, conventional till, grass and a conservation reserve program) on concentrations of 14 heavy metals and confirmed that land use has an impact on the concentration of heavy metals in topsoil.^64^ Therefore, appropriate land use practices can be helpful in remediating contaminated soils. Moreover, best agronomic practices and environmentally friendly farming approaches are becoming widely adopted and applied worldwide.^64^,^66^ Farming practices such as fallowing, crop rotation, mixed cropping, no-till, conventional till, grass and conservation farming strategies are aimed at reducing contamination of soils, but they have not been widely practiced in China. Planting of non-food crops such as cotton, flax, flowers, and ornamentals can improve soil quality, but this is not widely practiced in China due to a shortage of arable land.^21^ The study confirmed that land use had significant effects on As, boron (B), Cd, Co, Cu, Ni, and silicon (Si) concentration in soils.

Another alternative approach to mitigating heavy metal pollution is regular monitoring of crop fields to decrease exposure to major toxic elements.^12^,^34^,^53^,^55^ Furthermore, monitoring increased utilization of pesticides, fertilizers, and other agrochemicals is useful for mitigating pollution of soils and crops.

**Figure 10:**
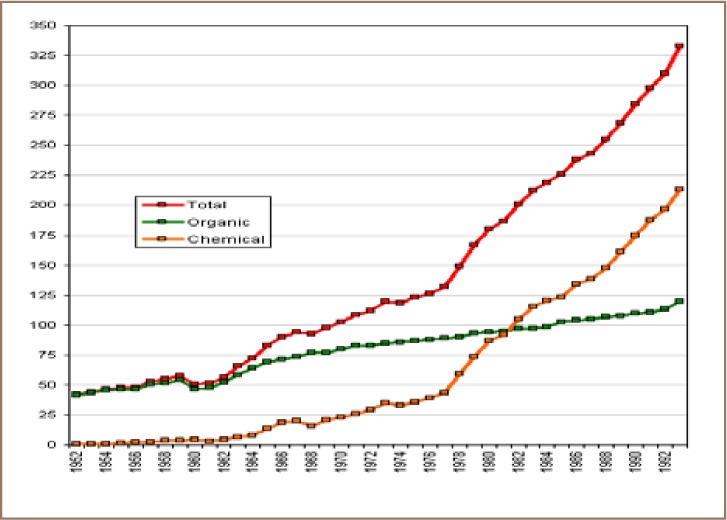
Chemical and organic fertilizer application in China, 1952 - 1993 (kg/ha).^67^

Agriculture in China is highly intensive and the use of chemical fertilizers has greatly increased, especially since the late 1970s (*Figure 10*). According to Heilig *et al.*, Chinese farmers used more chemical fertilizers than organic fertilizers on their fields in 1982 and this gap had nearly doubled by 1993.^67^ Similar to inorganic fertilizers, repeated application of pesticides in crop fields to control pests and weeds, especially in rice fields, has resulted in an increase of heavy metal elements in China.^68^ To that end, close monitoring of the application of agrochemicals and implementation of sustainable agriculture practices can help to mitigate the prevalent contamination of soils and crop by heavy metal elements.

### Bioremediation and Adoption of Sustainable Farming

Bioremediation is a soil reclamation approach that involves the use and rearing of soil biotas such as microorganisms, plants, and fungi that are able to bind, extract or transform contaminants in soil to create less toxic ecosystems.^43^ Bioremediation is often touted as a state-of-the-art technique used for heavy metal removal and/or recovery from polluted environments. The technique utilizes inherent biological mechanisms to eradicate hazardous contaminants using microorganisms and plants, or their products, to restore contaminated soil to its original condition. It is also an environmentally friendly and cost-effective technique for heavy metal removal/recovery compared to conventional chemical and physical techniques, which are often more expensive and ineffective, especially for low metal concentrations.

#### Phytoremediation and Cultivar Selection

Phytoremediation employs the use of green plants for the cleanup of contaminated environments. Metal uptake by plants is dependent on a number of factors such as plant species, soil chemistry, and metal species type. Studies confirm that differences in metal uptake between plants depend on nutrient requirements and growth conditions. This remediation method maintains the biological properties and physical structure of the soil. Although this technique is environmentally friendly, cost-effective, and offers the possibility of bio-recovery of metal contamination, it takes time. It involves phytoextraction, where plants remove metals from the soil by concentrating them in their harvestable parts, and phytostabilization, where plants reduce the mobility and bioavailability of pollutants by immobilization.^69–71^ Some plants concentrate metals in the roots, indicating that root surface ion exchange characteristics may present a barrier to metal adsorption into plant tissues. Others store high levels of metals in their leaves. For instance, cabbage, lettuce and tobacco uptake high levels of Cd and accumulate higher levels in their leaves than their roots or stems. It is therefore important to identify selected plants for remediation purposes based on the different adsorption characteristics of various plant species.

Bioavailability refers to the fraction of the total contaminant mass in soil and sediment available to receptor organisms, including human and ecological organisms. Metal availability to plants is a process strongly controlled by plant characteristics. Plant species control their rhizosphere by exudation of protons and organic acids via their roots, and during a vegetation cycle by modifying metal complexation via litter decomposition. These general patterns are further complicated by the symbiosis of higher plants with various types of mycorrhizal fungi. These fungi are able to modify metal availability to plant roots and may strongly modify metal toxicity.^69^ Many study findings have shown variation in the uptake and distribution of heavy metal elements among and within crop species that are commonly used as food, such as rice and vegetable crops.^72^ These crops remove a varying amount of heavy metal elements from soils due to their genetic variation.^56^,^58^,^69^ For example, Tang *et al.* stated that Indian mustard and sunflower had high uptake of Cd. Variation is present within varietals as well. For instance, japonica rice, which is more commonly grown in northeastern China, has a lower tendency to absorb cadmium than Indica, which is more common in the southern parts of China.^69^

#### Microbial Remediation

Microbial remediation involves the use of microorganisms for the absorption, precipitation, oxidation, and reduction of heavy metals in soils. Microorganisms have metabolic capabilities that allow them to utilize various toxic compounds through respiration, fermentation, and co-metabolism.^73^ For instance, fungi constitute a high proportion of the microbial biomass in soil. Their large surface-to-volume ratio and high metabolic activity are used to change the heavy metal dynamics in soil.^74^ Moreover, studies have reported that microorganisms are able to live a long time in heavy metal-contaminated soils, allowing sufficient time for bioaccumulation, biomineralization, biosorption, and biotransformation of elements in the soils. Owing to these abilities, they have been used as bio-sorbents for heavy metal removal and recovery of soils.^73^ Bacterial, algae and fungi bio-sorbents are commonly used for the removal of heavy metal pollutants from soils. In particular, algae and fungi are reported to be very effective in biosorption of heavy metal elements from soils. Mustapha *et al.* reported algae to be a more efficient bio-sorbent (with a reported rate of 15.3% to 84.6%) compared to other microbial bio-sorbents (bacteria and fungi).^75^ Additionally, both fungal and algae bio-sorbents are considered to be inexpensive and eco-friendly methods suitable for remediation of contaminated soils.

These and other bioremediation methods can be adapted to China because they are inexpensive, environmentally friendly and efficient. The implementation of one or two of these technologies may not be a complete solution for this complex problem, but using integrated remediation approaches is feasible as soil pollution in China involves multiple actors.

#### Biochar Treatment

Biochar is a product extracted mainly from solid waste and biomass of agricultural and forestry residues through a pyrolysis process.^62^,^76^ Many recent studies have found that applying biochar to soils has a high capability to mitigate heavy metal contamination due to the ability of biochar to adsorb heavy metal elements in soils. Moreover, biochar can improve soil fertility by enhancing the cation exchange rate due to its high pH value and reduce uptake of heavy metals by crops. Most of the biochar materials are alkaline in nature and hence they have a liming effect that may contribute to the reduction of the mobility of heavy metals in contaminated soils.^77^ However, the adsorption ability of biochar varies depending on the types of heavy metals and biochar type being employed.^78–80^ A summary of production methods and reduction of heavy metal contaminants through application of different types of biochar is provided below (Table 1).

**Table 6:** Effect of Biochar Application on Heavy Metals in Soils Across Different Studies

**Species**	TEQ^*^	ADI^*^	**ΣPCBs**	**% Fat**
*Bamboo*	Not available	Cd	Combined effect of electrokinetics, 79.6% removal of extractable Cd within 12 days	Wang et al.(2007)^81^
*Hardwood*	450°C	As, Cd, Cu, Zn	10-fold reduction in Cd in soil pore water; 300 and 45-fold reduction of Zn concentrations in column leaching tests	Beesley et al.(2011)^78^
*Hardwood*	450°C	As, Cd, Cu, Pb, Zn	Biochar surface mulch enhanced As and Cu mobility in the soil profile; little effect on Cd and Pb	Beesley et al.(2010)^82^
*Wood*	200°C & 400°C	Cd, Zn	>90% reduction in Zn and Cd leaching loss	Debela et al.(2010)^83^
*Peanut husks*	500°C	Cd	99.2% Cd reduction	Ryan et al.(2016)^80^
*Waste wood*	520°C	Cd	18% Cd reduction	
*Soybean stalks*	700°C	Hg	86.4% Hg reduction	

Application of biochar has been widely accepted as a potential solution for soil contamination. For instance, Bian *et al.* reported a decrease in grain Cd and Pb accumulation by applications of biochar due to its effect on soil pH.^84^ Therefore, this is another alternative option that can be implemented in China as it is a cheap and efficient approach to mitigating heavy metals in soils, especially in smallholder farming systems.

#### Lime and Compost Treatment

Studies confirm that metal uptake is higher in acidic soils due to the higher solubility of most metal species at lower pH, whereas cation adsorption is highest between pH 5 to 7 and anion adsorption is best under pH 6.2.^63^ According to many studies, adding lime and compost is a commonly used method to stabilize soil heavy metals. This approach is considered to be a very effective method for reduction of the bioavailability of heavy metals, as it is able to minimize uptake by plants. Singh *et al.* reported reductions of up to 62.1%, 64.4%, 71.9%, 62.1% and 58.9% in the bioavailability of Cu, Fe, Ni, Cd and Cr, and Zn and Pb, respectively, in lime-treated soils.^85^

The use of compost is another simple approach that has been proven to mitigate heavy metals from soils.^85^ At the same time, compost can help to replace intensive use of inorganic fertilizer which is an important source of contamination of farmland soils in China.

Furthermore, there have been promising research findings on the effectiveness of vermicomposting, which involves the use of worms to decompose wastes such as vegetables, food wastes and crop remains into vermicompost that can be used as organic fertilizer. Because of its binding capacity with metals, it is reported to increase uptake by plants and hence it can remediate soils affected by heavy metals. Dabke SV reported a clear decrease in concentrations of chromium in vermicompost-treated sites.^86^ Therefore, application of vermicomposts fertilizers can be a useful practice if adopted in the smallholder farming system in China. In one hand this practice can help to reduce the overuse of chemical fertilizers and support a sustainable farming system on the other hand may help to mitigate heavy metals from contaminated farmlands where application of other remediation approaches such as soil excavation and engineering may be less practical and expensive solutions.

## Conclusions and Recommendations

Contamination of soils by heavy metals is an important challenge facing many countries around the world, including China. Many studies have confirmed that the rates of soil contamination in some parts of China are above recommended standards. Generally, the present study found that about 10.18% of farmland soil, accounting for 13.86% of grain production, is affected by heavy metals, mainly Cd, Hg, Cu, and Ni. The major sources of heavy metal pollution in China are anthropogenic activities along with rapid urbanization and industrialization processes. The spatial distribution of heavy metal pollution in China is highly variable, and can be attributed to natural and human factors. However, provinces with intensive industrial activities are more affected than others. From the 14 most highly affected provinces, Henan, Shandong, and Sichuan rank in the top 3 in terms of contamination of farmland soil by heavy metals. In addition, over 60% of cropland is located in these affected provinces, illustrating the need for appropriate monitoring of crops produced in these regions. Close follow-up of rice and vegetable crops produced in China is needed for the development of sustainable and feasible approaches to mitigate potential crop contamination and associated health risk issues. To that end, a comprehensive monitoring framework was launched by the Chinese government that aims to determine sound reclamation strategies. Remediation strategies of soils affected by contamination in general and heavy metals in particular can be more holistic if they incorporate systematic, dynamic and integrated approaches that are feasible, sustainable and are able to accommodate different actors. Such strategies need to accommodate not only the efforts of governments, universities and research institutions, but also should involve the general public and industries. Particularly, as smallholder farmers are the most vulnerable group in most of cases to the impact of soil contamination, they should play a key role in the formulation and implementation of mitigation strategies. Moreover, technological and other approaches are required in order to achieve sustainability. Approaches such as mitigation of emissions and close monitoring of sources of heavy metals such as mining, smelting and other metal consuming industries are very important steps to combatting soil pollution with heavy metals. Land use management and monitoring of agriculture, application of biochar, lime and compost (such as vermicompost) and bioremediation are less costly alternative approaches to the reclamation of contaminated soil. Therefore, mitigation strategies require coordination of different actors and integration of all feasible reclamation approaches to remediate contaminated soils.
