# Supplementary material for: Review of the Spatial Distribution, Source and Extent of Heavy Metal Pollution of Soil in China: Impacts and Mitigation Approaches
Source: J Health Pollut. 2018 Mar 12;8(17):53–70. doi: 10.5696/2156-9614-8.17.53 (PMC6221442; doi:10.5696/2156-9614-8.17.53)
Supplement: Supplementary file 1 [file i2156-9614-8-17-53.s1.pdf]

Supplemental Material 1.

Summary of Heavy Metals Pollution by Study Area

City/Location	Determined Elements								Reference
	As	Cd	Cr	Cu	Hg	Ni	Pb	Zn	
31 metropolises in China	✓	✓	✓	✓	✓	✓	✓	✓	Cheng *et al.* (2014) [3]
Aquatics ecosystems in China	✓	✓	✓	✓	✓	✓	✓	✓	Wang, *et al.* (2016)[52]
Baoji			✓	✓		✓	✓	✓	Cheng *et al.* (2014) [3]
Beijing		✓	✓	✓		✓	✓	✓	Cheng *et al.* (2014) [3]
Beijing				✓			✓	✓	Qingjie *et al.* (2008) [2]
Beijing Metropolis		✓	✓	✓		✓	✓	✓	Sun *et al.* (2016) [6]
Changchun	✓	✓	✓	✓	✓		✓	✓	Cheng *et al.* (2014) [3]
Changsha	✓	✓	✓	✓	✓		✓	✓	Cheng *et al.* (2014) [3]
Chongqing	✓	✓	✓	✓	✓	✓	✓	✓	Cheng *et al.* (2014) [3]
Cities in China					✓				Wu *et al.* (2006)[14]
Dabaoshan mine, southern China		✓		✓			✓	✓	Zhuang *et al.* (2016)[48]
Dongting Lake	✓	✓	✓	✓	✓		✓		Li F, *et al.* (2013)[34]
Dushanzi, Xinjiang	✓	✓	✓	✓		✓	✓	✓	Wang, *et al.* (2016)[9]
Eastern China		✓	✓	✓		✓	✓	✓	Tang *et al.* (2014)[59]
Farmland Soil in China					✓				Zhong *et al.* (2016)[39]
Fuxin City in Northeast China	✓	✓	✓	✓		✓	✓	✓	Chen, *et al.* (2015)[16]
Fuzhou	✓	✓	✓	✓	✓	✓	✓	✓	Cheng *et al.* (2014) [3]
Guangzhou		✓		✓		✓	✓		Cheng *et al.* (2014) [3]
Guiyang		✓		✓		✓	✓	✓	Cheng *et al.* (2014) [3]
Hangzhou		✓	✓	✓		✓	✓	✓	Cheng *et al.* (2014) [3]
Hefei		✓	✓	✓			✓		Cheng *et al.* (2014) [3]
Huainan city, Anhui, East China	✓	✓	✓	✓	✓		✓		Ying, *et al.* (2016)[12]
Huizhou City, Guangdong Province		✓		✓			✓	✓	Li *et al.* (2011)[46]
Hunan Province	✓	✓	✓		✓	✓	✓		Zeng *et al.*(2015)[56]
Lake Dalinouer, China		✓		✓			✓	✓	Hou *et al.* (2013)[36]
Lanzhou				✓			✓	✓	Cheng *et al.* (2014) [3]
Qinghai Haibei		✓							Ye *et al.* (2011)[11]
Quanzhou Bay		✓				✓			Zhao *et al.* (2012)[22]
Several cities in China	✓	✓	✓	✓	✓	✓	✓	✓	Wei *et al.* (2010)[10]
Shanghai		✓	✓	✓		✓	✓	✓	Cheng *et al.* (2014) [3]
Shenyang in Northeast China	✓	✓	✓	✓	✓		✓	✓	Li, *et al.* (2013) [34]
Shijiazhuang	✓	✓	✓	✓	✓		✓	✓	Cui, *et al.* (2011)[45]
Shuangtaizi estuary, Northern Bohai Bay		✓		✓			✓	✓	Li C, *et al.* (2017)[51]
Southwestern China**	✓	✓	✓	✓	✓	✓	✓	✓	Cheng *et al.* (2014)[31]
Taiyuan City, China	✓	✓	✓	✓	✓	✓	✓	✓	Liu *et al.* (2014)[17]
Western Xiamen Bay and vicinity		✓	✓	✓		✓	✓	✓	Zhang *et al.* (2007)[49]
Xinjiang City	✓	✓	✓	✓		✓	✓	✓	Wang *et al.* (2016) [9]
Yucheng City, Shandong Province	✓	✓	✓	✓	✓	✓	✓	✓	Jia *et al.*(2010)[68]
Zhejiang	✓	✓			✓		✓		Huang *et al.* (2013)[13]
Total	**19**	**34**	**26**	**33**	**18**	**23**	**35**	**29**	

** Along with 68 other elements
